# Use of transcriptomics and co-expression networks to analyze the interconnections between nitrogen assimilation and photorespiratory metabolism

**DOI:** 10.1093/jxb/erw170

**Published:** 2016-04-25

**Authors:** Carmen M. Pérez-Delgado, Tomás C. Moyano, Margarita García-Calderón, Javier Canales, Rodrigo A. Gutiérrez, Antonio J. Márquez, Marco Betti

**Affiliations:** ^1^Departamento de Bioquímica Vegetal y Biología Molecular, Facultad de Química, Universidad de Sevilla, C/ Profesor García González, 1, 41012-Sevilla, Spain; ^2^Departamento de Genética Molecular y Microbiología, Facultad de Ciencias Biológicas, FONDAP Center for Genome Regulation, Millennium Nucleus Center for Plant Systems and Synthetic Biology, Pontificia Universidad Católica de Chile, Santiago 8331150, Chile; ^3^Instituto de Bioquímica y Microbiología, Facultad de Ciencias, Universidad Austral de Chile, Campus Isla Teja s/n, Valdivia 5090000, Chile

**Keywords:** Co-expression networks, *Lotus*, *japonicus*, nitrogen metabolism, nitrogen nutrition, photorespiration, transcriptomics.

## Abstract

Nitrogen is one of the most important nutrients for plants and, in natural soils, its availability is often a major limiting factor for plant growth. Here we examine the effect of different forms of nitrogen nutrition and of photorespiration on gene expression in the model legume *Lotus japonicus* with the aim of identifying regulatory candidate genes co-ordinating primary nitrogen assimilation and photorespiration. The transcriptomic changes produced by the use of different nitrogen sources in leaves of *L. japonicus* plants combined with the transcriptomic changes produced in the same tissue by different photorespiratory conditions were examined. The results obtained provide novel information on the possible role of plastidic glutamine synthetase in the response to different nitrogen sources and in the C/N balance of *L. japonicus* plants. The use of gene co-expression networks establishes a clear relationship between photorespiration and primary nitrogen assimilation and identifies possible transcription factors connected to the genes of both routes.

## Highlight

A clear interconnection between photorespiration and primary nitrogen assimilation is established in *Lotus japonicus*, and key transcription factors connected to both routes are identified using transcriptomics and gene co-expression networks.

## Introduction

Nitrogen is one of the most important nutrients for plants, and, in natural soils, its availability is often a major limiting factor for plant growth. The use of nitrogen by plants involves several steps, including uptake, assimilation, translocation, and different forms of recycling and remobilization processes, all of them of crucial importance in terms of nitrogen utilization efficiency (Hirel *et al.*, 2007; Masclaux-Daubresse *et al.*, 2010). Primary nitrogen assimilation by plants involves the use of different forms of inorganic nitrogen (NO_3_^−^ and/or NH_4_^+^), depending on nitrogen availability, plant species, and adaptations. Alternatively, symbiosis with bacteria enables some plant species, most notably legumes, to use atmospheric N_2_ that is reduced to NH_4_^+^ in the nodules by the action of bacterial nitrogenase. In addition, efficient secondary NH_4_^+^ assimilation must also exist in plants in order to reassimilate the NH_4_^+^ ions that can be produced endogenously in the plants from processes such as photorespiration, phenylpropanoid biosynthesis, or amino acid catabolism (Betti *et al.*, 2014).

The NH_4_^+^ generated by the photorespiratory nitrogen cycle can be up to 20 times that resulting from the reduction of NO_3_^−^ (Canvin, 1990; Guo *et al.*, 2007). The large amounts of NH_4_^+^ generated by the photorespiratory cycle are produced in the mitochondria as a result of the conversion of two molecules of glycine into one molecule of serine by the combined action of glycine decarboxylase (GDC; EC 2.1.2.10) and serine hydroxymethyl transferase (SHMT; EC 2.1.2.1). NH_4_^+^ is transported to the chloroplast where it is reassimilated by the plastidic glutamine synthetase and ferredoxin-glutamate synthase cycle (GS2/Fd-GOGAT). Several important pathways such as nitrogen assimilation, respiration, one-carbon metabolism, purine biosynthesis (Bauwe *et al.*, 2010), and redox signaling (Foyer *et al.*, 2009) interact in different ways with photorespiration. Moreover, it has been described that the conditions that inhibited photorespiration (elevated CO_2_) also strongly inhibited NO_3_^−^ assimilation in C_3_ plants (Rachmilevitch *et al.*, 2004; Bloom, 2015). Photorespiration serves as a mechanism for plants to use NO_3_^−^ as a nitrogen source without diverting energy from CO_2_ fixation. The linkage between photorespiration and NO_3_^−^ assimilation provides higher plants with a relatively abundant nitrogen source that other organisms cannot afford to use, but that C_3_ plants can use with little additional energy cost (Bloom, 2015).

Furthermore, the form of nitrogen available to plants can affect their time and rate of seed germination, leaf expansion and function, dry matter partitioning between shoot and root, and root architecture (Andrews *et al.*, 2013). Despite the fact that more energy is needed for the assimilation of NO_3_^−^, most plants prefer NO_3_^−^ over NH_4_^+^. With the exception of NH_4_^+^-tolerant species, NH_4_^+^ as a sole nitrogen source, in addition to internal production of NH_4_^+^ by processes such as photorespiration (Keys *et al.*, 1978), may prove toxic to the plant. Indeed, in comparison with NO_3_^−^ uptake, much less is known about the regulatory mechanisms controlling either NH_4_^+^ or N_2_ acquisition (Ruffel *et al.*, 2008). Notably, the toxic effect of external NH_4_^+^ can be relieved by co-provision of NO_3_^−^, so-called mixed nutrition (NH_4_NO_3_) (Forde and Clarkson, 1999). Also, a fascinating and still poorly understood aspect of nitrogen nutrition is that in most cases the growth of a plant on NH_4_NO_3_ can surpass the maximal growth compared with either NO_3_^−^ or NH_4_^+^ alone. This relief of NH_4_^+^ toxicity by NO_3_^−^ may be related to a synergism between the signaling routes of NH_4_^+^ and NO_3_^−^ (Britto and Kronzuker, 2002).

*Lotus japonicus* is a temperate legume that can grow using the atmospheric N_2_ fixed in the nodules or by using external sources of nitrogen, such as NO_3_^−^ and/or NH_4_^+^. The utilization of NO_3_^−^ requires its reduction to NH_4_^+^ from the consecutive action of nitrate reductase (NR; EC 1.7.1.1/2) and nitrite reductase (NiR; EC 1.7.7.1) enzymes. Then, the NH_4_^+^ synthesized as a result of both primary and secondary assimilation is assimilated into glutamine and then into glutamate by the enzymes glutamine synthetase (GS; EC 6.3.1.2) and glutamate synthase (GOGAT; EC 1.4.7.1 or EC 1.4.1.14) (Márquez *et al.*, 2005). Different sets of GS and GOGAT isoforms exist in plants which are specifically associated with different processes such as primary NO_3_^−^ or NH_4_^+^ assimilation, N_2_ fixation, and/or secondary NH_4_^+^ reassimilation. In particular, plastidic GS2 and Fd-GOGAT have been reported to be the crucial enzymes for the reassimilation of photorespiratory NH_4_^+^ thanks to the isolation of photorespiratory mutants deficient in GS2 (Wallsgrove *et al.*, 1987) or Fd-GOGAT (Somerville and Ogren, 1980).

The first GS photorespiratory mutants isolated from legume plants were identified several years ago in our laboratory from the model legume *L. japonicus* (Orea *et al.*, 2002; Márquez *et al.*, 2005). These mutants were shown to be specifically deficient in GS2 and have been substantially characterized at the molecular and physiological levels (Orea *et al.*, 2002; Márquez *et al.*, 2005; Betti *et al.*, 2006, 2012*a*, 2014; García-Calderón *et al.*, 2012), including recent transcriptomics and metabolomics studies (Díaz *et al.*, 2010; Betti *et al.*, 2012a; Pérez-Delgado *et al.*, 2013). Under a CO_2_-enriched atmosphere, where photorespiration is suppressed, the mutants did not show any visible phenotype (Orea *et al.*, 2002).

The objective of this study is to determine the effect of different forms of nitrogen nutrition and the effect of photorespiration on gene expression in order to analyze the possible interconnections among these processes in *L. japonicus* plants. For this purpose, a comparative transcriptomic study was carried out using wild-type (WT) plants and plants with a deficiency in GS2 (*Ljgln2-2*) grown with different nitrogen regimes (NO_3_^−^, NH_4_^+^, NH_4_NO_3_, or under conditions of biological nitrogen fixation) and in different photorespiratory conditions. Furthermore, a set of gene co-expression networks was built to study the connection between photorespiratory genes and genes of primary nitrogen assimilation, with the ultimate goal of identifying regulatory candidate genes co-ordinating these processes.

We first demonstrate that several important transcriptomic changes occurred in leaves of *L. japonicus* when plants were cultivated with different nitrogen sources, including genes involved in nitrogen, carbon, and secondary metabolism. To study the possible interconnections between primary nitrogen assimilation and photorespiration, WT and *Ljgln2-2* mutant plants were examined under different forms of nitrogen nutrition and different photorespiratory conditions. The data obtained provide novel information on the possible role of plastidic GS2 in the response to different nitrogen sources and on the C/N balance of *L. japonicus* plants. Finally, co-expression networks were built using the nitrogen- and photorespiration-responsive genes previously identified. A clear interconnection between nitrogen assimilation and photorespiration was established in *L. japonicus*, and several key transcription factors that could be involved in the co-ordinate regulation of these metabolic routes were identified.

## Materials and methods

### Growth conditions and harvesting of plant material

*Lotus japonicus* (Regel) Larsen cv. Gifu (B-129-S9) was initially obtained from Professor Jens Stougaard (Aarhus University, Aarhus, Denmark) and then self-propagated at the University of Seville. The *Ljgln2-2* mutant, which lacks GS2 protein and activity (Betti *et al.*, 2006), was isolated from the photorespiratory mutant screening carried out using ethyl methanesulfonate as described previously (Orea *et al.*, 2002). The mutant progeny of two consecutive backcrosses into the WT background were used. WT and mutant seeds were scarified and surface-sterilized, then germinated in 1% agar in Petri dishes and transferred to pots using vermiculite as solid support. Five seedlings were planted in each pot and grown until plants had seven trefoils in a growth chamber under 16 h:8h day:night, 20:18 °C, with a photosynthetic photon flux density of 250 μmol m^−2^ s^−1^ and a constant humidity of 70%. When required, CO_2_ was automatically injected to a final concentration of 0.7% (v/v) (high CO_2_ atmosphere).

Nodulated plants were inoculated with *Mesorhizobium loti* and watered with nitrogen-free ‘Hornum’ medium supplemented with 3mM KCl (Handberg and Stougaard, 1992). *Mesorhizobium loti* TONO JA76 (Kawaguchi *et al.*, 2002) was grown in YM liquid medium (Vincent, 1970) at 28 °C to an optical density at 600 nm=1, and then collected by centrifugation for 30min at 2408 *g* and resuspended in 0.75% (w/v) NaCl. Once sown in the pots, the plants were inoculated by the addition of 2ml of this bacterial suspension.

Plants under different forms of nitrogen nutrition were watered with ‘Hornum’ nutrient solution containing 10mM KNO_3_ (NO_3_^−^ plants), 10mM NH_4_Cl supplemented with 3mM KCl (NH_4_^+^ plants), or 5mM NH_4_NO_3_ supplemented with 3mM KNO_3_ (NH_4_NO_3_ plants). The nutrient solutions were renewed every 3 d. These nutritional conditions were used taking into consideration the recommended growth conditions for *L. japonicus* (Handberg and Stougaard, 1992; Orea *et al,*. 2005). Moreover, previous works have shown that the concentrations of the different nitrogen sources used here are not toxic for *L. japonicus* (Orea *et al.*, 2005). After all the plants had reached the same size (an average of seven trefoils), leaf tissue was harvested. All the leaf samples used in this work were harvested 4h after the beginning of the light period. The stage of seven trefoils was reached after ~38, 35, 36, or 42 d in the case of plants growing under symbiotic conditions or with NH_4_NO_3_, NO_3_^−^, or NH_4_^+^ alone, respectively. Every harvest involved at least three independent biological replicates for each genotype and treatment. A biological replicate consisted of tissue pooled from five plants grown in the same pot.

Plants were grown continuously either under normal CO_2_ atmosphere (0.04% v/v), to permit normal rates of photorespiration, or under high CO_2_ (0.7% v/v) atmosphere in order to suppress photorespiration and to permit the normal growth of the *Ljgln2-2* mutant.

### RNA extraction and qRT–PCR

Leaf material was flash-frozen in liquid nitrogen, homogenized with a mortar and pestle, and kept at −80 °C until use. Three independent biological replicates were used for the quantitative real-time reverse transcription–PCR (qRT–PCR) analysis. Total RNA was isolated using the hot borate method (Sánchez *et al.*, 2008). The integrity and concentration of the RNA preparations were checked using an Experion bioanalyzer (Bio-Rad) with RNA StdSens chips and a Nano-Drop ND-1000 (Nano-Drop Technologies), respectively.

For qRT–PCR analysis, total RNA was treated with the TURBO DNA-free DNase (Ambion). Reverse transcription was carried out using SuperScript III reverse transcriptase (Invitrogen), oligo(dT), and RNAsin RNase inhibitor (Ambion). DNA contamination and RNA integrity were checked by carrying out qRT–PCRs with oligonucleotides that amplified an intron in the *LjHAR1* gene and the 3' and 5' ends of *L. japonicus* glyceraldehyde-3-phosphate dehydrogenase, respectively. qRT–PCRs were carried out in 10 μl in a Lightcycler 480 thermal cycler (Roche) using a SensiFAST SYBR No-ROX Kit (Bioline). Expression data were normalized using the geometric mean of four housekeeping genes: *LjGPI*-anchored protein (probeset chr3.CM0047.42), *LjPp2A* (probeset chr2.CM0310.22), *LjUBC10* (probeset chr1.TM0487.4), and *LjUBQ4* (probeset chr5.CM0956.27) that were selected amongst the most stably expressed genes in plants (Czechowski *et al.*, 2004). A list of all the oligonucleotides used is provided in Supplementary Table S1 at *JXB* online.

### DNA chip hybridization and data analysis

Two independent biological replicates were used for the transcriptomic analysis of plants grown in different nitrogen sources. Microarray slides were designed and produced using Agilent eArray (Agilent Technologies; http://www.agilent.com) specifically developed for *L. japonicus*, and hybridized with total leaf RNA according to the manufacturer’s instructions.

The microarrays were scanned, the raw image files were processed, and data analysis was performed by Agilent Corporation. MIAME compliant data are deposited at Array Express (http://www.ebi.ac.uk/arrayexpress) as E-MTAB-4177. Data were normalized using the function rma of the limma package (Linear Models for Microarray Data, v. 2.10.5) (Smyth, 2004) of bioconductor.

The differentially expressed genes of plants grown under different forms of nitrogen nutrition were determined with a one-way ANOVA using a false discovery rate (FDR) threshold of *P*<0.01 based on the 10 000 permutation test with the MeV module of the TM4 package (http://www.TM4.org) (Saeed *et al.*, 2003) followed by a post-hoc Tukey test with R (http://www.R-project.org/). Hierarchical clustering of transcriptomic data was carried out with Expander software version 7.1 (Shamir *et al.*, 2005; Ulitsky *et al.*, 2010) using average linkage.

Differentially expressed genes between WT and *Ljgln2-2* mutant plants and between photorespiratory and non-photorespiratory conditions were determined using Rank products (Breitling *et al.*, 2004) passing an FDR threshold of *P*<0.1. The differentially expressed genes were visualized using the MapMan program (Usadel *et al.*, 2005) and analyzed according to the corresponding metabolic pathways or functional categories using Pathexpress (Goffard and Weiller, 2007). The default threshold of *P*<0.1 and FDR rate correction was used for Pathexpress.

Gene sequences were obtained at the Kazusa database (http://www.kazusa.or.jp/lotus/).

### Co-expression network construction

#### Microarray data collection and pre-processing

Microarray data used in this work were obtained from the experiments published by Sánchez *et al.* (2008, 2011), Høgslund *et al.* (2009), Díaz *et al.* (2010), Betti *et al.* (2012b), and Pérez-Delgado *et al.* (2013). These experiments have a total of 240 hybridizations. CEL files of these experiments are available in the public microarrays database EBI (https://www.ebi.ac.uk/arrayexpress/). Code numbers of experiments are: E-MEXP-1204, E-TABM-715, E-MEXP-2344, E-MEXP-2690, E-MEXP-1726, E-MEXP-3710, and E-MEXP-3603. Background correction and normalization of the raw data sets were performed using Robust MultiChip Analysis (RMA) implemented in the ‘affy’ R package (Gautier *et al.*, 2004).

#### Identification of differentially expressed genes

The non-parametric Rank product method (Breitling *et al.*, 2004) was used to identify differentially expressed genes between treatment and control conditions using an FDR threshold of *P*<0.05.

#### Network construction

The network construction was developed according to Canales *et al.* (2014). A weighted gene co-expression network was constructed using the WGCNA R package version 1.27.1 (Langfelder and Horvath, 2008) with differentially expressed genes. First, the Pearson correlation matrix was weighted by raising it to a power (β). To choose the appropriate power, the network topology for various soft-thresholding powers was evaluated using pickSoftThreshold function and β=6 was chosen because this ensured an approximate scale-free topology of the resulting network. Next, the pairwise measure of gene co-expression of the resulting weighted network was transformed into a topological overlap (TO) similarity measure, which is a robust measure of pairwise interconnectedness (Yip and Horvath, 2007). A TO similarity measure between two genes (ij) is defined as: *T0ij*=Σ*u aiuauj*+*aij* min (*ki*,*kj*)+1–*aij* where *ki*=*u aiu* was the node connectivity and *a* is the network adjacency. Finally, the co-expression network was visualized using Cytoscape v 3.0 and analyzed using the NetworkAnalyser plugin (Doncheva *et al.*, 2012). In order to simplify the display of the network and to focus on relevant relationships, only edges in this network of the corresponding TO similarity measure above a threshold of 0.11 are presented in this work. Furthermore, the NetworkAnalyser plugin was used to assess which genes in the network form hubs.

## Results and Discussion

### Transcriptomics of *L. japonicus* leaves from plants grown in different nitrogen sources

Previous studies indicated that the expression levels of different key genes for nitrogen metabolism and photorespiration were affected by a defect in the photorespiratory cycle (Pérez-Delgado *et al.*, 2013, 2015). In addition, it has been shown that the rate of photorespiration strongly influences the efficiency with which plants can use different nitrogen sources such as NO_3_^−^ or NH_4_^+^ (Bloom, 2015). Therefore, in the present study, we examined the transcriptomic response of *L. japonicus* plants to different forms of nitrogen nutrition, as a first step to determine if the regulation of primary nitrogen assimilation and of photorespiration may be interconnected in legumes. The study was carried out in leaves because this is where the photorespiratory cycle is active. Special attention was paid to the genes encoding transcription factors whose expression was altered under the different conditions considered.

**Fig. 1.**
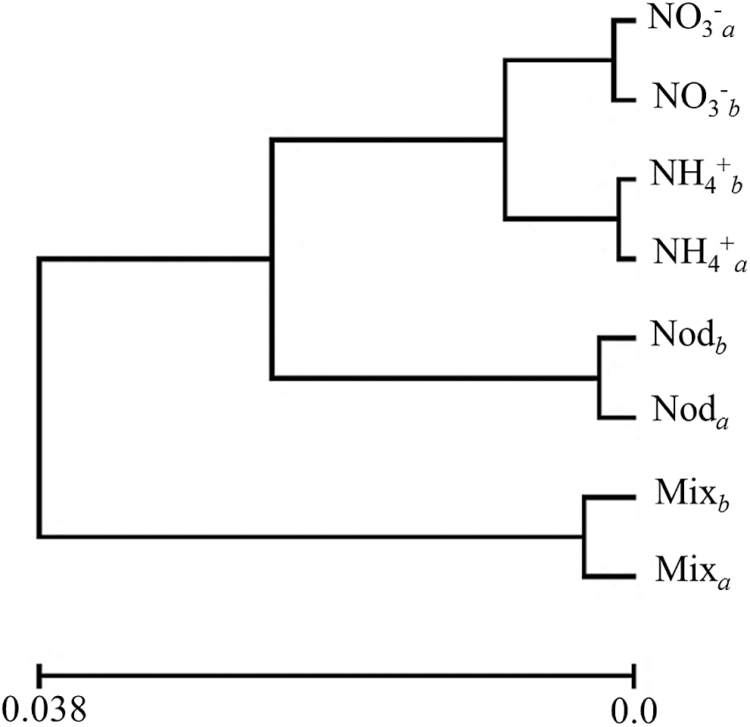
Hierarchical clustering of the different nitrogen nutrition treatments according to the probesets differentially expressed in these conditions as determined by ANOVA using an FDR threshold of *P*<0.01. Plants were cultivated under four different nitrogen regimens: under symbiotic conditions (Nod) or with NH_4_NO_3_ (Mix), NO_3_^−^ only, or NH_4_^+^ only. The clustering analysis was carried out with the Expander software using average linkage. *a* and *b* indicate the biological replicates of the samples in the microarray (E-MTAB-4177).

**Fig. 2.**
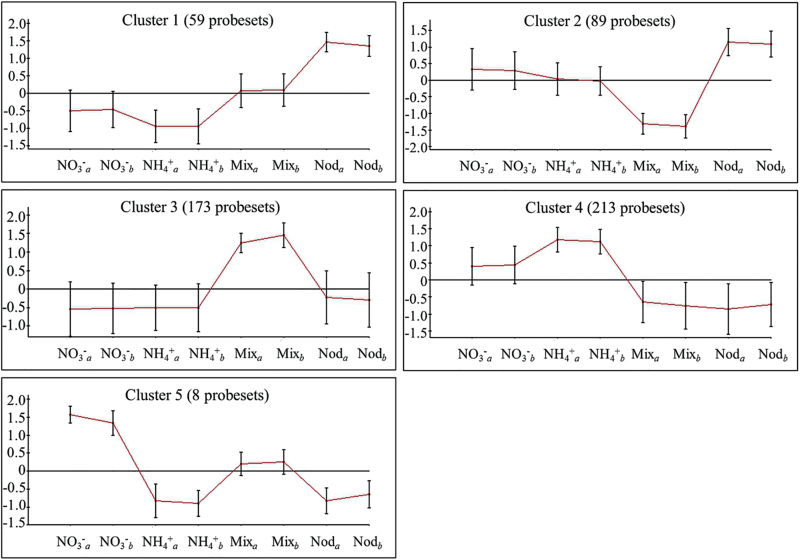
Hierarchical clustering of the probesets differentially expressed among plants cultivated with different forms of nitrogen nutrition according to the ANOVA carried out. The different nitrogen conditions used were: purely symbiotic conditions (Nod), NH_4_NO_3_ (Mix), NO_3_^−^ only, or NH_4_^+^ only. The analysis was carried out with the Expander software and the clusters were determined using average linkage and a distance threshold of 0.3. For each cluster, the average log_2_ of the fold change in expression of all the differentially expressed probesets under each form of nitrogen nutrition is represented. The number of probesets in each cluster is indicated in parentheses. *a* and *b* indicate the biological replicates of the samples in the microarray (E-MTAB-4177). (This figure is available in colour at *JXB* online.)

*Lotus japonicus* plants were grown with different mineral nitrogen sources (NO_3_^−^, NH_4_^+^, or NH_4_NO_3_) or under conditions of biological nitrogen fixation (Nod). A comparative transcriptomic analysis was carried out in leaves of plants grown under the four different nitrogen regimes. Genes that showed significantly different levels of expression among treatments were identified by performing a one-way ANOVA together with a Tukey test (further details are given in the Materials and methods). The *P*-values were corrected for multiple testing using an FDR method with a cut-off of 0.01. A total of 542 probesets were found to be differentially expressed in plants cultivated with the different nitrogen treatments. A global clustering analysis of the whole data set indicated that the transcriptional responses to NO_3_^−^ or NH_4_^+^ were very similar (Fig. 1). Moreover, plants growing with either NO_3_^−^ or NH_4_^+^ clustered more closely to the plants grown under purely symbiotic conditions (Nod) than to the plants grown with NH_4_NO_3_, indicating that the co-provision of NO_3_^−^ and NH_4_^+^ triggers a unique transcriptional response compared with the other nitrogen regimes. A hierarchical clustering of the genes that showed significantly different levels of expression between treatments was carried out based on their levels of expression under each nitrogen condition. Five different clusters were obtained according to this analysis (Fig. 2) and are analyzed hereafter. A complete list of the genes and of the transcription factors present in each cluster can be found in Supplementary Tables S2 and S3, respectively.

#### Cluster 1

This first cluster contains genes that were more expressed in nodulated plants compared with the other nutritional conditions. Several genes for the biosynthesis of phenolic compounds were found among this group. 4-Coumarate:CoA ligase (probeset chr4.CM0061.26), which catalyzes one of the first common steps for the biosynthesis of all phenolic compounds, was more expressed in nodulated plants. Genes for the synthesis and modification of lignin precursors such as cinnamoyl-CoA reductase (probesets Ljwgs_051770.2 and chr5.CM0200.51) and caffeoyl CoAO-methyltransferase (probeset Ljwgs_030453.1) were also present in this cluster together with a gene for isoflavonoid biosynthesis (isoflavone reductase, probeset chr2.CM0249.94). Phenolic compounds are a large family of secondary metabolites involved in different processes, including plant–pathogen interactions, pollination, light screening, seed development, and allelopathy. Moreover, several of these compounds show an important antioxidant capacity (Hernández *et al.*, 2008). Different genes for phenolic metabolism were found to be altered in their expression levels under different growing conditions in *L. japonicus* plants, some of which were also connected to photorespiration (García-Calderón *et al.*, 2015). The higher expression of genes for the biosynthesis of phenolic compounds in nodulated plants confirms previous results that showed the existence of important differences in redox metabolism in nodulated plants compared with those cultivated with NO_3_^−^ (M. García-Calderón *et al*., unpublished). Also, it is well known that flavonoids and isoflavonoids have a key role in nodulation (Falcone Ferreyra *et al.*, 2012) and this may also explain the higher expression levels of these genes in nodulated plants. Finally, only one transcription factor belonging to the ERF (ethylene response factor) subfamily was found in this group of genes (probeset TM1489.5) (see Supplementary Table S3).

#### Cluster 2

In a similar way to cluster 1, the 89 probesets of this group corresponded to genes showing the highest levels of expression in nodulated plants; but, in addition to that, their expression levels were lowest under NH_4_NO_3_ nutrition. In agreement with the analysis of cluster 1, several genes involved in the biosynthesis of phenolic compounds were more expressed in nodulated plants, such as the genes encoding a 4-coumarate:CoA ligase isoform (probeset Ljwgs_064187.1) and enzymes involved in flavonoid biosynthesis such as flavanone 4-hydroxylase (probeset Ljwgs_024122.1) and anthocyanidin reductase (chr1.CM0579.2), again suggesting an increased requirement for this class of secondary metabolites in nodulated plants. The only transcription factor present in this group is a zinc finger protein of the C2H2 type (probeset TM0489.21.1) of unknown function (see Supplementary Table S3).

#### Cluster 3

The 173 probesets in this group corresponded to genes with higher expression in plants growing under NH_4_NO_3_ nutrition compared with the other three nutritional conditions. This group included genes encoding enzymes of both nitrogen and carbon metabolism. Key enzymes for amino acid metabolism such as lysine-ketoglutarate dehydrogenase (probeset chr4.CM0343.10) and asparagine synthetase (probesets gi897772 and TM1307.9.1) were more expressed under NH_4_NO_3_ nutrition. On the other hand, genes encoding enzymes of central carbon metabolism were also present in this cluster, such as phosphoenolpyruvate carboxykinase (probeset Ljwgs_107668.1), phosphoenolpyruvate carboxylase (probeset chr5.CM0311.22), and enolase (probeset chr1.TM0356.3), together with genes involved in the metabolism of storage polysaccharides such as fructans (β-fructofuranosidase, probesets Ljwgs_038389.1.1 and Ljwgs_012880.1). Eleven transcription factors were highly expressed in NH_4_NO_3_-fed plants compared with the other nutritional forms (Supplementary Table S3). While none of these factors has been characterized in *L. japonicus*, some interesting information can be obtained by searching for their orthologous genes in Arabidopsis. Probesets chr1.CM0088.102 and chr1.CM0375.38 are orthologs to the Arabidopsis transcription factors ATML1, a HUA-like transcription factor family member, and to AXR2, respectively, all of them involved in embryo and shoot development (Takada *et al.*, 2013; Jali *et al.*, 2014; Sato *et al.*, 2015). Two NAC transcription factors (probesets Ljwgs_045720.1 and Ljwgs_055404.1) correspond to Arabidopsis VNI2 and NTL9, which regulate leaf longevity and senescence (Yoon *et al.*, 2008; Yang *et al.*, 2011). These transcription factors may be related to the fact that the growth of plants cultivated with NH_4_NO_3_ can surpass the maximal growth compared with either NO_3_^−^ or NH_4_^+^ alone (Orea *et al.*, 2005).

#### Cluster 4

The 213 probesets in this group corresponded to genes with higher levels of expression under NH_4_^+^ nutrition (and, to a minor extent, under NO_3_^−^ conditions) compared with NH_4_NO_3_ nutrition and purely symbiotic conditions. Very few genes coding for enzymes of central metabolic pathways, with the exception of an NO_3_^−^ transporter and a potassium transporter, were found in this cluster. Ten genes related to the response to different kinds of abiotic or biotic stress were induced by NH_4_^+^ nutrition, which may be justified by the higher levels of oxidative stress that are normally observed in plants growing with NH_4_^+^ as the sole nitrogen source (Britto and Kronzuker, 2002). On the other hand, a lot of genes related to the modification of chromatin, RNA transcription and translation, and protein post-translational modification showed higher expression mainly in plants grown with NH_4_^+^. These included several genes coding for histones, DNA helicases, RNA helicases, RNases, and several RNA-binding proteins, proteins involved in the regulation of translation, post-translational modification of proteins, protein degradation (proteases, ubiquitin-conjugation enzymes and components of the proteasome), and protein targeting different organelles. Moreover, several transcription factors clustered in this group (see Supplementary Table S3). Some of them were related to cell and tissue development, such as the orthologs of Arabidopsis LZF1 (probeset Ljwgs_049567.1), BSM (probeset Ljwgs_142668.1), and ATRNJ (probeset Ljwgs_035337.1) that are required for the biogenesis of mitochondria and chloroplasts (Chang *et al.*, 2008; Quesada *et al.*, 2011; Chen *et al.*, 2015) or the orthologs of Arabidopsis RSM1, AGL62, PIF3, and CDF4 (probesets TM1490.11, chr5.CM0180.16.1, chr5.CM0048.61, and chr4.CM0399.23, respectively) that are involved in the morphogenesis of different plants tissues (see Hamaguchi *et al.*, 2008; Roszak and Köhler, 2011; Wang *et al.*, 2014; Pi *et al.*, 2015, respectively, for more information on these transcription factors). In addition, some of the transcription factors found in this cluster are involved in the generation of circadian rhythms. This included the ortholog to the core clock protein TOC1 (probeset Ljwgs_147347.1) (Strayer *et al.*, 2000) as well as other proteins involved in the circadian system such as the orthologs to the JMJD5 protein (probeset Ljwgs_035693.2) (Jones *et al.*, 2010) and the ARR3 protein (probeset chr2.CM0028.12) (Salomé *et al.*, 2006). Interestingly, probeset chr5.CM0909.32 encodes the transcription factor OBF4 (also called TGA4), whose ortholog gene (At5g10030) has been associated with the response to NO_3_^−^ (Álvarez *et al.*, 2014) and NH_4_^+^ (Patterson *et al.*, 2010) in Arabidopsis. These data suggest that the leaf response to NH_4_^+^ nutrition is mediated mainly by reorganization of gene expression and cell function rather than through the modification of central metabolic routes.

#### Cluster 5

The expression of the genes corresponding to the eight probesets of this group was mainly different between NO_3_^−^-fed and NH_4_^+^-fed plants. With the exception of a serine protease, these genes encoded proteins of unknown function. This is in good agreement with the data from Fig. 1 that indicate that there are few transcriptional differences in the leaves of plants grown with these two nitrogen sources.

In summary, transcriptomic data analysis showed that different groups of genes were differentially expressed in leaves of *L. japonicus* depending on the nitrogen source provided. Under purely symbiotic conditions, a higher expression of genes related to the biosynthesis of phenolic compounds, flavonoids, and isoflavonoids was observed, probably reflecting the importance of these secondary metabolites in the rhizobial symbiosis process. On the other hand, plants growing under NH_4_NO_3_ nutrition showed changes in genes of primary metabolism. An induction of genes involved in carbon and nitrogen metabolism was observed in plants growing with NH_4_NO_3_ (cluster 3). It is well known that NO_3_^−^ functions as a signal molecule to induce the transcripts and activities of enzymes related to NH_4_^+^ assimilation and organic acid synthesis providing carbon skeletons for amino acid synthesis (Scheible *et al.*, 1997; Wang *et al.*, 2004). These results indicate the existence of important differences in the link between carbon and nitrogen metabolism under the different forms of nitrogen nutrition examined. Therefore, it was of great interest to explore further the possible interconnections of processes mainly related to carbon metabolism such as photorespiration and nitrogen assimilation. Interestingly, 40 transcription factors responsive to the different nitrogen regimes were identified, suggesting that they could have a relevant function in the different nitrogen signaling or metabolic processes involved.

### Interconnection of photorespiration and nitrogen assimilation: studies with *Ljgln2-2* mutants

**Fig. 3.**
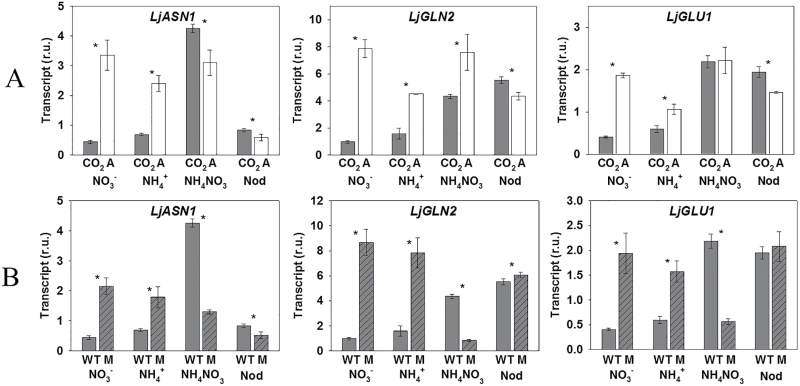
(A) Expression levels of some key genes of nitrogen metabolism in WT plants under a CO_2_-enriched atmosphere (CO_2_, gray bars) or normal air (A, white bars) and under different nitrogen conditions: purely symbiotic conditions (Nod), NH_4_NO_3_, NO_3_^−^ only, or NH_4_^+^ only. (B) Expression levels of the same genes in WT (gray bars) and *Ljgln2-2* plants (M, gray striped bars) grown under the same different forms of nitrogen nutrition and CO_2_-enriched atmosphere. *LjASN1*, asparagine synthetase 1; *LjGLN2*, plastidic glutamine synthetase; *LjGLU1*, ferredoxin-dependent GOGAT. Data are the mean ±SD of three independent biological replicates. *Indicates a significant difference between high CO_2_ and normal air conditions in (A) and between the WT and *Ljgln2-2* in (B) as determined by Student’s test (*P*<0.05).

In order to analyze the possible interactions between the assimilation of different nitrogen sources and photorespiration, we first compared the levels of expression of key genes for nitrogen assimilation in plants grown under different nitrogen regimes, maintained either under normal photorespiratory conditions (normal CO_2_ atmosphere) or under photorespiratory-suppressed conditions (high CO_2_ atmosphere). For reasons of simplicity, the results obtained for some representative genes (*LjASN1*, *LjGLN2*, and *LjGLU1*) are presented in Fig. 3A, but the whole data set can be found in Supplementary Fig. S1. The levels of atmospheric CO_2_ had a significant influence on the levels of expression of several nitrogen-assimilatory genes (Fig. 3A; Supplementary Fig. S1). It has to be taken into consideration that the impact of atmospheric CO_2_ on gene expression may be due to its effects on the photorespiratory cycle and/or to the high availability of carbon under high CO_2_ conditions. In addition, the patterns of expression of the nitrogen-assimilatory genes in the different nitrogen sources were different in plants cultivated in photorespiratory-suppressed conditions and in plants cultivated in active photorespiratory conditions. These results indicated that the atmospheric CO_2_ concentration/photorespiration affect the response of *L. japonicus* plants to different nitrogen sources. A good agreement was found between the gene expression levels measured by qRT–PCR and the expression levels measured by microarray for the WT plants under normal CO_2_ conditions and under nutrition with different nitrogen forms (Supplementary Table S4).

The key enzyme at the interface between carbon and nitrogen metabolism is GS since this enzyme incorporates the NH_4_^+^ resulting from primary and secondary nitrogen assimilation processes to yield glutamine. In particular, the plastidic GS2 isoform is the enzyme in charge of photorespiratory NH_4_^+^ reassimilation in plants, and it is therefore the main point of connection between nitrogen assimilation and photorespiratory metabolism. For this reason, the influence of the nitrogen source on the levels of expression of different nitrogen-assimilatory genes was also studied in the photorespiratory mutant *Ljgln2-2* (that lacks the plastidic GS2 enzyme) in comparison with the WT. Both WT and mutant plants were grown under high CO_2_ conditions in order to permit the normal growth of the mutant. Again, for reasons of simplicity, the results obtained for some representative genes (*LjASN1*, *LjGLN2*, and *LjGLU1*) are presented in Fig. 3B, but the whole data set can be found in Supplementary Fig. S2. The patterns of expression of the nitrogen-assimilatory genes in mutant plants in the different nitrogen sources were different from the patterns of expression of these genes in the WT plants cultivated in the same nitrogen sources. These results indicated that the lack of GS2 affects the response of *L. japonicus* plants to different nitrogen sources.

On the other hand, a more integrative analysis of all the expression data of nitrogen assimilation genes obtained in WT and *Ljgln2-2* mutant plants in different photorespiratory conditions shows that there was a close similarity in the differential levels of gene expression of WT plants in high CO_2_ versus air treatments (Fig. 3A) compared with WT versus *Ljgln2-2* mutant plants in high CO_2_ (Fig. 3B) under the different nitrogen forms of nutrition examined. This suggests that the deficiency in GS2 has a similar effect on the transcript levels of nitrogen-assimilatory genes as the decrease of CO_2_ concentration and the presence of active photorespiratory conditions in the WT plants under the different forms of nitrogen nutrition studied.

It has been examined whether other genes, in addition to key genes for nitrogen assimilation, could be modulated by changes in CO_2_ concentration/photorespiratory conditions and/or the lack of GS2. For this purpose, a complete transcriptomic analysis was carried out using the available microarray data from the experiments published by Díaz *et al.* (2010) and Betti *et al.* (2012*b*). The transcriptional changes produced in the WT by the decrease of the CO_2_ atmospheric concentration were compared with the transcriptional changes produced by plastidic GS deficiency in plants grown under non-photorespiratory conditions (high CO_2_).

**Fig. 4.**
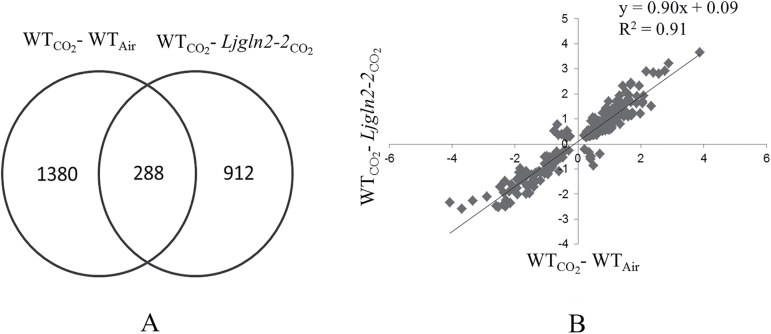
(A) Venn diagram showing the number of probesets modulated by the decrease of CO_2_ concentration and/or the absence of plastidic GS (*P*<0.1 and FDR correction). (B) Comparison of the fold change values for the probesets that are significantly elicited by both conditions.

A Rank product analysis with FDR correction (*P*<0.1) identified 1668 differentially expressed probesets when comparing WT plants grown in high CO_2_ or in normal air conditions, which represent genes that can be modulated by carbon and/or active photorespiratory conditions. On the other hand, 1200 differentially expressed probesets as a result of the lack of GS2 under non-photorespiratory conditions were identified using the same statistical cut-off. Interestingly, 24% of these probesets (288) were common to the two transcriptome comparisons carried out (Fig. 4A; Supplementary Table S5). Moreover, these probesets showed a strong linear correlation (*r* ^2^=0.91) between the levels of their corresponding fold change in gene expression in the two transcriptomic analyses, with a slope of the regression line of 0.9 that indicates that the transcriptional changes were always very similar between the two transcriptomic comparisons carried out (Fig. 4B). Only 18 of the 288 common probesets showed an opposite response to the decrease of CO_2_ concentration and to the lack of GS2. All these results indicate the existence of an important number of genes whose expression levels changed in the same way in response to the lack of GS2 and to the decrease of the atmospheric CO_2_ concentration in addition to the genes for nitrogen metabolism that were studied previously in this work.

The MapMan and PathExpress tools (Usadel *et al.*, 2005; Goffard and Weiller, 2007) were utilized to analyze in more detail the above-mentioned group of 288 probesets (Supplementary Fig. S3). The MapMan program allows the visualization of the changes observed in transcriptomic data by providing an overview of metabolic pathways. Visualization of the 288 probesets using this software indicated that the corresponding genes were apparently evenly distributed among the different routes of primary and secondary metabolism (Supplementary Fig. S3). However, PathExpress permitted the identification of four significantly over-represented metabolic pathways within this group of genes. The routes for flavonoid biosynthesis, histidine metabolism, d-arginine and d-ornithine metabolism, and starch and sucrose metabolism were significantly over-represented within the 288 probesets. Moreover, pyruvate decarboxylase (probeset Ljwgs_007060.2), a key gene of central carbon metabolism that has also been reported to have a role in stress tolerance (Pinhero *et al.*, 2011), was found in this group together with other genes related to carbon metabolism encoding a sugar transporter (probeset Ljwgs_136027.1), a diacylglycerol kinase (probeset Ljwgs_067758.1), and an α-galactosidase (probeset Ljwgs_025958.1), amongst others. In addition, several genes related to redox function were modulated such as glutathione *S*-transferase (probesets Ljwgs_037557.1 and Ljwgs_027688.1), cytochrome P450 (probesets chr1.CM0591.58 and Ljwgs_099719.2), and glutaredoxin (probeset chr1.CM0109.23.1), suggesting that there is an interconnection of carbon, photorespiration, and the lack of GS2 with redox metabolism or stress responses. It is very interesting to note that there was a modulation of a glycolate oxidase (*LjGO1*) gene (probeset Ljwgs_013523.1). This gene may have a role in stress responses in *L. japonicus* in relation to photorespiratory NH_4_^+^ accumulation (Pérez-Delgado *et al.*, 2013). Previous studies showed that several genes differentially expressed in *Ljgln2-2* and WT plants under non-photorespiratory conditions were also elicited by drought stress specifically in *Ljgln2-2*, thus confirming the existence of a relationship between the lack of GS2 and the stress-responsive machinery in *L. japonicus* (Díaz *et al.*, 2010).

The present study has shown first that the lack of GS2 affects the response of *L. japonicus* plants to different nitrogen sources. Secondly, a novel link between the lack of plastidic GS2 and the decrease in external CO_2_ provided to the plants was observed, which is supported by the finding of 288 commonly modulated probesets in both types of situations. Therefore, it appears that a relationship between the lack of GS2 and carbon metabolism must exist in *L. japonicus* plants. Previous work from our group has established how a GS2 defect in nitrogen assimilation affects carbon metabolism in this plant (Betti *et al.*, 2012*a*; García-Calderón *et al.*, 2012; Pérez-Delgado *et al.*, 2013). Finally, six transcription factors were also found in the group of 288 commonly modulated probesets (Supplementary Table S6). These transcription factors may be important for the C/N balance and for the photorespiratory metabolism of *L. japonicus* plants in relation to GS2 and were further investigated together with the nitrogen-responsive transcription factors that were previously identified in the first section of the paper using co-expression networks as is shown below.

### Use of co-expression networks to study the interconnection between photorespiration and nitrogen assimilation

To explore further the interaction between photorespiration and primary nitrogen assimilation, a gene co-expression network was constructed using the WGCNA R package and the transcriptomic data available for *L. japonicus.* The use of gene co-expression networks was proved to be a very useful tool for the prediction of network driver genes and for the identification of transcription factors with a role in the control of gene expression in nitrogen metabolism (Vidal *et al.*, 2010; Canales *et al.*, 2014). This new analysis integrates the data obtained in the previous experiments of this study with the aim of determining if the genes for nitrogen assimilation and photorespiratory metabolism are interconnected and to search for regulatory genes that may co-ordinate these processes. The co-expression networks generated were visualized using the Cytoscape software and analyzed using the NetworkAnalyser plugin (Doncheva *et al.*, 2012).

**Fig. 5.**
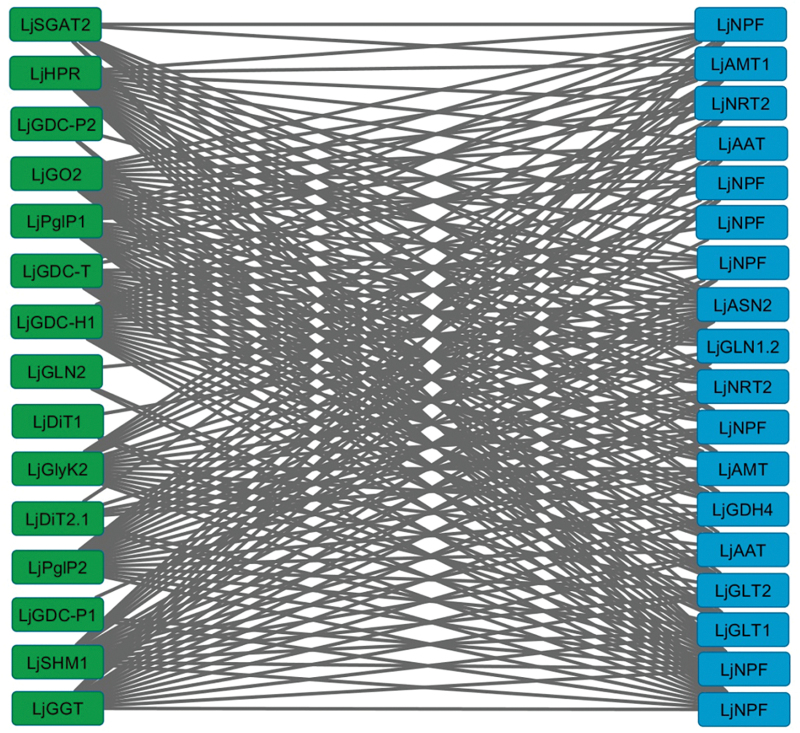
Co-expression network analysis of the connections detected among photorespiratory genes (rectangles in the left-hand column) and genes of primary nitrogen assimilation (rectangles in the right-hand column). Edges represent predicted regulatory interactions between target genes. The genes present in the network image are: phosphoglycolate phosphatase (*LjPglP1* and *LjPglP2*); glycine decarboxylase (*LjGDC-H1*, *LjGDC-P1*, *LjGDC-P2*, and *LjGDC-T*); glycerate kinase (*LjGlyK2*); glutamate:glyoxylate aminotransferase (*LjGGT*); serine:glyoxylate aminotransferase (*LjSGAT2*); serine hydroxymethyltransferase (*LjSHMT1*); glycolate oxidase (*LjGO2*); hydroxypyruvate reductase (*LjHPR*); plastidic glutamine synthetase (*LjGLN2*); plastidic dicarboxylate transporter (*LjDiT1* and *LjDiT2.1*); NO_3_^−^ transporter (*LjNPF*); NH_4_^+^ transporter (*LjAMT*); asparagine synthetase (*LjASN2*); glutamate dehydrogenase (*LjGDH4*); cytosolic glutamine synthetase (*LjGLN1.2*); NADH-dependent glutamate synthase (*LjGLT1* and *LjGLT2*); and aspartate aminotransferase (*LjAAT*). (This figure is available in colour at *JXB* online.)

A first co-expression network was constructed using the genes for primary nitrogen assimilation and for photorespiratory metabolism (Supplementary Table S7). This analysis identifies the most connected genes between the two groups across multiple experiments (Fig. 5; Supplementary Table S8). Eighteen genes for primary nitrogen assimilation were clearly connected to 15 photorespiratory genes. Among the genes of nitrogen metabolism, we found seven low affinity NO_3_^−^ transporters (*LjNPF*), two high affinity NO_3_^−^ transporters (*LjNRT2*), two NH_4_^+^ transporters (*LjAMT* and *LjAMT1*), one asparagine synthetase (*LjASN2*), one glutamate dehydrogenase (*LjGDH4*), one cytosolic glutamine synthetase (*LjGLN1.2*), two NADH-dependent glutamate synthases (*LjGLT1* and *LjGLT2*), and two aspartate aminotransferases (*LjAAT*). This result suggests that primary nitrogen assimilation is connected to photorespiration through NO_3_^−^ and NH_4_^+^ transporters and NH_4_^+^ assimilatory genes. Among the photorespiratory genes that were connected to genes of primary nitrogen assimilation, we found two phosphoglycolate phosphatases (*LjPglP1* and *LjPglP2*), four glycine decarboxylases (*LjGDC-H1*, *LjGDC-P1*, *LjGDC-P2*, and *LjGDC-T*), one glycerate kinase (*LjGlyK2*), two aminotransferases (*LjGGT* and *LjSGAT2*), one serine hydroxymethyltransferase (*LjSHMT1*), one glycolate oxidase (*LjGO2*), hydroxypyruvate reductase (*LjHPR*), plastidic glutamine synthetase (*LjGLN2*), and two chloroplast envelope transporters (*LjDiT1* and *LjDiT2.1*). The vast majority of the different photorespiratory genes were connected to some extent to primary nitrogen assimilation. These results confirm the existence of a clear connection between primary nitrogen assimilation and photorespiration, as suggested also by different authors using other types of approaches (Rachmilevitch *et al.*, 2004; Pérez-Delgado *et al.*, 2013, 2015; Bloom, 2015). Other co-expression networks were generated as a control in order to evaluate the connection between photorespiratory genes and genes of other pathways. The connection between photorespiratory genes and genes of nucleotide synthesis, cellulose synthesis, and genes for DNA repair and cell division was very low, indicating that the connection between photorespiration and nitrogen metabolism was significant (Supplementary Table S9).

**Fig. 6.**
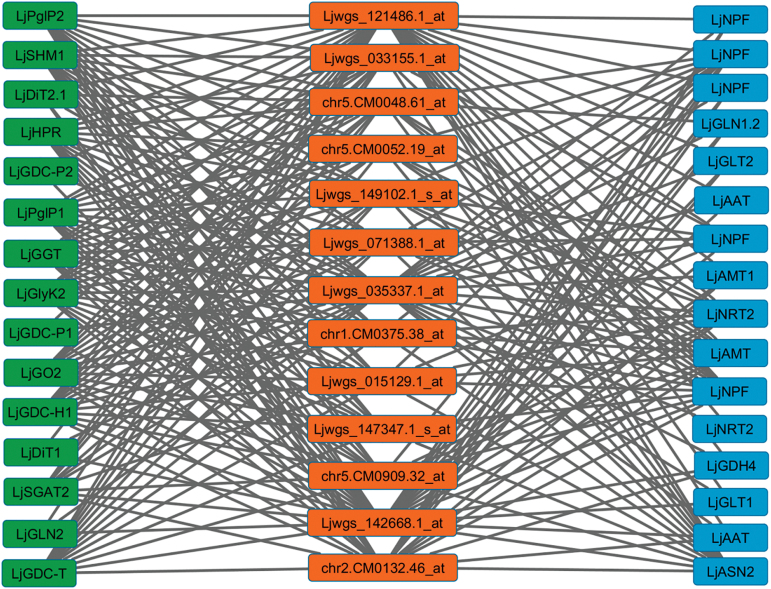
Co-expression network analysis of the connections detected among photorespiratory genes (rectangles in the left-hand column) and genes of primary nitrogen assimilation (rectangles in the right-hand column) with transcription factor genes (rectangles in the central column). Edges represent predicted regulatory interactions between transcription factors and target genes. The photorespiratory genes and genes of primary nitrogen assimilation present in the network image are: phosphoglycolate phosphatase (*LjPglP1* and *LjPglP2*); glycine decarboxylase (*LjGDC-H1*, *LjGDC-P1*, *LjGDC-P2*, and *LjGDC-T*); glycerate kinase (*LjGlyK2*); glutamate:glyoxylate aminotransferase (*LjGGT*); serine:glyoxylate aminotransferase (*LjSGAT2*); serine hydroxymethyltransferase (*LjSHMT1*); glycolate oxidase (*LjGO2*); hydroxypyruvate reductase (*LjHPR*); plastidic glutamine synthetase (*LjGLN2*); plastidic dicarboxylate transporter (*LjDiT1* and *LjDiT2.1*); NO_3_^–^ transporter (*LjNPF*); NH_4_^+^ transporter (*LjAMT*); asparagine synthetase (*LjASN2*); glutamate dehydrogenase (*LjGDH4*); cytosolic glutamine synthetase (*LjGLN1.2*); NADH-dependent glutamate synthase (*LjGLT1* and *LjGLT2*); and aspartate aminotransferase (*LjAAT*). Transcription factors are represented by their probeset. (This figure is available in colour at *JXB* online.)

A second co-expression network was constructed using the genes of primary nitrogen assimilation and photorespiratory metabolism and also different transcription factor genes from *L. japonicus*, obtained in the available databases (Jin *et al.*, 2014) (see Supplementary Table S10). A total of 370 transcription factors were connected to at least one gene for primary nitrogen assimilation and to at least one photorespiratory gene (Supplementary Table S11). A high percentage of these genes belong to the families ERF, basic helix–loop–helix (bHLH), and Myb. To distill from this analysis the candidate regulatory genes that may co-ordinate primary nitrogen assimilation and photorespiration, a third co-expression network was built from the previous one using only the transcription factors that were modulated by either the nitrogen source (Supplementary Table S3) or the GS2 deficiency and the variation in atmospheric CO_2_ concentration (Supplementary Table S6). Thirteen transcription factors connected to both photorespiration and nitrogen assimilation were identified in this analysis (Table 1; Fig. 6; Supplementary Table S12).

**Table 1.** Transcription factors (TFs) connected to genes of primary nitrogen assimilation and to photorespiratory genes using a co-expression networks analysis Transcription factors highlighted with an asterisk were also modulated by the transfer from photorespiratory suppressed conditions to active photorespiratory conditions.

**Probeset**	**TF family**	**Number of connections**	**Gene (Kazusa 3.0**)	**Ortholog gene in Arabidopsis**
Ljwgs_121486.1_at	Unknown	31	Lj2g3v1984810.1	At4g17800
Ljwgs_035337.1_at*	Trihelix	31	Lj0g3v0261399.1	At5g63420
Ljwgs_142668.1_at*	mTERF	30	Lj2g3v2197630.1	At4g02990
chr2.CM0132.46_at	bHLH	23	Lj2g3v1984450.1	At4g37850
Ljwgs_149102.1_s_at	Unknown	17	Lj4g3v3015070.1	At4g12750
chr5.CM0048.61_at	bHLH	17	Lj5g3v1533330.1	At1g09530
Ljwgs_015129.1_at*	bHLH	16	Lj3g3v0028580.1	At2g22770
chr5.CM0909.32_at*	bZIP	15	Lj5g3v1697630.1	At5g10030
Ljwgs_071388.1_at*	Myb-related	15	Lj4g3v0973380.1	At4g39250
chr1.CM0375.38_at*	Unknown	14	Lj1g3v4450520.1	At3g23050
chr5.CM0052.19_at*	ERF	14	Lj5g3v1937400.1	At5g64750
Ljwgs_033155.1_at	bHLH	13	Lj0g3v0136069.1	At1g32640
Ljwgs_147347.1_s_at*	ARR	11	Lj4g3v1658890.2	At5g61380

Two of the transcription factors identified (probesets Ljwgs_121486.1_at and Ljwgs_035337.1_at) are of particular interest because they are connected to all the photorespiratory genes and primary nitrogen assimilation genes in the co-expression network. The probeset Ljwgs_035337.1_at encodes a transcription factor whose ortholog gene in Arabidopsis (At5g63420) plays a vital role in embryo morphogenesis and apical-basal pattern formation by regulating chloroplast development (Chen *et al.*, 2015), whereas the function of the gene encoded by the probeset Ljwgs_121486.1_at is unknown. The genes encoding both transcription factors were more expressed in plants growing with NH_4_^+^ (cluster 4 from Fig. 2). Probeset Ljwgs_142668.1_at is connected to all the photorespiratory genes and almost all the nitrogen genes, and encodes a transcription factor whose ortholog gene in Arabidopsis is required for the biogenesis of mitochondria and chloroplasts (Quesada *et al.*, 2011). The corresponding gene was also more expressed in plants growing with NH_4_^+^ (cluster 4). In addition, the probesets chr2.CM0132.46_at, Ljwgs_149102.1_s_at, and chr5.CM0048.61_at also showed a high number of connections to both photorespiratory and primary nitrogen assimilation genes. chr2.CM0132.46_ at and Ljwgs_149102.1_s_at encode transcription factors of unknown function, while chr5.CM0048.61_at encodes PIF3, a bHLH transcription factor whose ortholog gene in Arabidopsis (At1g09530) is involved in morphogenesis (Wang *et al.*, 2014). Probesets Ljwgs_015129.1_at, chr5.CM0909.32_at, and Ljwgs_071388.1_at are connected to different NO_3_^−^ transporters, one NH_4_^+^ transporter, and one asparagine synthetase, and to 10 different photorespiratory genes. Ljwgs_015129.1_at corresponds to a bHLH transcription factor whose ortholog gene in Arabidopsis (At2g22760) is involved in the regulation of the formation of an endoplasmic reticulum-derived structure, the ER body (Matsushima *et al.*, 2004). The probeset chr5.CM0909.32_at encodes the transcription factor OBF4 (also named TGA4 transcription factor), whose ortholog gene in *A. thaliana* (At5g10030) has been associated with the NO_3_^−^ response (Álvarez *et al.*, 2014) and NH_4_^+^ response (Patterson *et al.*, 2010). Ljwgs_071388.1_at corresponds to a Myb-related transcription factor whose ortholog gene in Arabidopsis (At4g39250) is involved in plant development (Zhang *et al.*, 2013). Finally, it was also found that another four transcription factors were connected to several photorespiratory genes as well as to NO_3_^−^ transporters and to an asparagine synthetase (probesets Ljwgs_033155.1_at and Ljwgs_147347.1_s_at), and also to one NH_4_^+^ transporter (chr1.CM0375.38_at and chr5.CM0052.19_at). chr1.CM0375.38_at is a transcription factor whose ortholog gene in Arabidopsis (AXR2, At3g23050) is involved in embryo and shoot development (Sato *et al.*, 2015). The probesets chr5.CM0052.19_at and Ljwgs_033155.1_at encode an ERF and a bHLH factor, respectively, whose orthologous genes in Arabidopsis (At5g64750 and At1g32640, respectively) are involved in the response to abscisic acid and abiotic stress (Pandey *et al.*, 2005; De Ollas *et al.*, 2015). The ortholog to probeset Ljwgs_147347.1_s_at is the core clock protein TOC1, involved in the generation of circadian rhythms (Shimizu *et al.*, 2015).

The functional significance of the transcription factors identified in relation to photorespiration was further confirmed by the fact that eight of the 13 genes described here (highlighted with an asterisk in Table 1) were also modulated by the transfer from photorespiratory-suppressed conditions to active photorespiratory conditions (as can be revealed by analyzing the data from Pérez-Delgado *et al.,* 2013). Of particular interest is the probeset chr5.CM0052.19_at, whose expression levels were modulated up to 8-fold when plants were transferred to active photorespiratory conditions. Furthermore, two of the transcription factors detected with this co-expression analysis (probesets Ljwgs_015129.1_at and Ljwgs_033155.1_at) were also identified among the transcription factors that were commonly affected by the decrease of CO_2_ concentration and the lack of GS2 (Supplementary Table S6).

The results obtained in this work demonstrate the existence of multiple interconnections between primary nitrogen assimilation and photorespiration in *L. japonicus* plants, and allowed us to identify several transcription factors that could have a crucial role in the joint regulation of these processes. Further work would still be required to obtain mutants from the transcription factors identified and to determine their exact physiological significance.

### Conclusions

In this work it has been shown that several important transcriptomic changes occur in leaves of *L. japonicus* when plants are cultivated with different nitrogen sources. This includes genes involved in nitrogen, carbon, and secondary metabolism, as well as several transcription factors that may play an important role in nitrogen signaling or metabolism. In addition, the study of the *Ljgln2-2* mutants provides novel insights into the effects of the lack of plastidic GS in the response to different nitrogen sources and also shows the existence of a common transcriptomic response due to the lack of GS2 and to the changes in external CO_2_/photorespiratory activity, thus emphasizing the importance of GS2 in the C/N balance in *L. japonicus* plants. Finally, the use of co-expression networks allowed us to establish a clear interconnection between photorespiration and primary nitrogen assimilation and to identify several candidate genes for the co-ordination of these metabolic routes.

## Supplementary data

Supplementary data are available at *JXB* online.

Table S1. Primer sequences used for qRT–PCR measurements.

Table S2. Differentially expressed genes in plants grown with different nitrogen sources (NO_3_^−^, NH_4_^+^, NH_4_NO_3_, and nodulated plants) divided into different clusters.

Table S3. Differentially expressed transcription factor genes in plants grown with different nitrogen sources (NO_3_^−^, NH_4_^+^, NH_4_NO_3_, and nodulated plants) divided into different clusters.

Table S4. Validation of microarray and qRT–PCR data.

Table S5. Genes elicited by both the decrease of CO_2_ concentration and the lack of GS2.

Table S6. Transcription factors elicited by both the decrease of CO_2_ concentration and the lack of GS2.

Table S7. List of the genes for primary nitrogen assimilation and photorespiration.

Table S8. Co-expression network analysis of photorespiratory genes and genes of primary nitrogen assimilation.

Table S9. Control co-expression analysis of photorespiratory genes and genes of nitrogen metabolism, nucleotide synthesis, cellulose synthesis, and genes for DNA repair and cell division. Groups of genes were taken from MapMan.

Table S10. List of *Lotus japonicus* transcription factors available in the current databases.

Table S11. Co-expression network analysis of photorespiratory genes, genes for primary nitrogen assimilation, and transcription factors.

Table S12. Co-expression network analysis of photorespiratory genes, genes for primary nitrogen assimilation, and the transcription factors that were previously detected as responsive to nitrogen nutrition or changes in the photorespiratory conditions (from Supplementary Tables S3 and S6).

Figure S1. Expression levels of some key genes for nitrogen metabolism in WT plants under a CO_2_-enriched atmosphere (CO_2_) or normal air (A) and different nitrogen sources.

Figure S2. Expression levels of some key genes of nitrogen metabolism in WT and *Ljgln2-2* mutant plants (M) grown with different forms of nitrogen nutrition and under a CO_2_-enriched atmosphere.

Figure S3. MapMan overview of general metabolism and Pathexpress analysis of over-represented pathways for the 288 probesets that were modulated by both the decrease of CO_2_ concentration and the lack of plastidic GS.

Supplementary Data
