# Supplementary material for: Use of transcriptomics and co-expression networks to analyze the interconnections between nitrogen assimilation and photorespiratory metabolism
Source: J Exp Bot. 2016 Apr 25;67(10):3095–108. doi: 10.1093/jxb/erw170 (PMC4867901; doi:10.1093/jxb/erw170)
Supplement: Supplementary Data [file supp_67_10_3095__index.html]

Use of transcriptomics and co-expression networks to analyze the interconnections between nitrogen assimilation and photorespiratory metabolism — Use of transcriptomics and co-expression networks to analyze the interconnections between nitrogen assimilation and photorespiratory metabolism — Supplementary Data 

# Use of transcriptomics and co-expression networks to analyze the interconnections between nitrogen assimilation and photorespiratory metabolism

## Supplementary Data

Data files

- Supplementary\_table\_S1\_Figures\_S1\_S3.pdf - Supplementary Data
- Supplementary\_table\_S2.xls - Supplementary Data
- Supplementary\_table\_S3.xls - Supplementary Data
- Supplementary\_table\_S4.xls - Supplementary Data
- Supplementary\_table\_S5.xls - Supplementary Data
- Supplementary\_table\_S6.xls - Supplementary Data
- Supplementary\_table\_S7.xls - Supplementary Data
- Supplementary\_table\_S8.xls - Supplementary Data
- Supplementary\_table\_S9.xls - Supplementary Data
- Supplementary\_table\_S10.xls - Supplementary Data
- Supplementary\_table\_S11.xls - Supplementary Data
- Supplementary\_table\_S12.xls - Supplementary Data
