# Supplementary material for: Use of transcriptomics and co-expression networks to analyze the interconnections between nitrogen assimilation and photorespiratory metabolism
Source: J Exp Bot. 2016 Apr 25;67(10):3095–108. doi: 10.1093/jxb/erw170 (PMC4867901; doi:10.1093/jxb/erw170)
Supplement: Supplementary Data [file supp_erw170_Supplementary_table_S1_Figures_S1_S3.pdf]

Use of transcriptomics and co-expression networks to analyze the interconnections between nitrogen assimilation and photorespiratory metabolism. *CM Pérez-Delgado, TC Moyano, M García-Calderón, J Canales, RA Gutiérrez, AJ Márquez and M Betti*.

Supplementary Table S1. Primer sequences for qRT-PCR measurements.

Gene	Primers	
	**Forward**	**Reverse**
LjHAR1	TCATTTTCCATATTGGTCCTTCG	GAATTGTTCCTGTAATGGGTTTGG
LjGAPDH5'	AAGGATCGGGCGTTTGG	AGCAACAAGTTCAACATCGTCTCT
LjGAPDH3'	CGGTTACACTGAAGATGATGTGG	GATACTTGACCTGTTGTCGCCA
LjGPI-ap	AGGTTGTTCCGTGAATTTCG	GGTCCTTTGCATTTGCTTGT
LjPp2A	TGAGCTATGTGAAGCTGTTGGT	CAGCCTCATTATCACGCAGTAG
LjUBC10	GCTCTTATCAAGGGACCATCAG	ACTGCTCTGGAACAGAAAAAGC
LjUBQ4	TTCACCTTGTGCTCCGTCTTC	AACAACAGCACACACAGACAATCC
LjGLN1.1	TGGACCACAGGGCCCATAC	AATGTCACGCCCATAGGCTTT
LjGLN1.2	TGAGGTGTGGGTTGCTCGTT	AAGGACCACCCCAGCAATCT
LjGLN1.3	TAACCTCTCCGAGACCACCG	CCAATCCATATGTATTCGGCG
LjGLN1.4	AGAGAGACTGAGAAAGATGGAAAATGT	CAGGCCTCCTCTGTCCTCAA
LjGLN1.5	GGGTAGGCAGGGAGACTGAAA	GAAGCTGGCCTCCTGTCCTC
LjGLN2	GGAAGAGGGAGGCTTTGAGGT	CTGGTGGCGAAGGGATAGATT
LjGLU1	ATTCAACGAGTAACAGCGCCA	CATGGGCTTCAATAAGGCTTCTC
LjGLT1	TGGTGCTGATGCTATATGCCC	GTCAACCTGCAGTCGCCAA
LjGLT2	TTGGCAATGTGGCACTGTATG	GCTGCCATCCCGTTGAAAT
LjGDH1	GGAGATGTGCAAAACCCATGA	CGGTTAACTCCCAGGGTGAAA
LjGDH2	ACATGACAATGCTCGTGGTCC	AGGGTCAACCTCAGGGTGGTA
LjGDH3	GCTGCTGATGTGAAAGCGAAA	GCATCTGGGTCAGTGGGATG
LjGDH4	AAGGTTCAAACATGCCCTGCAC	AGCAGGAGCAACGAGAACATTAGC
LjASN1	TGGAGGACCAACTGTTGCATG	AGACCAAGCAGCATCCCACTC
LjASN2	TCAGTGAGCAAAGGTGTTGAACC	CAAGAGGAGAAACTTCCATCTTGG
LjASN3	CGAACTGGCAGTGATTGTGAAGTG	ATGCCAATAGCATCACGAGCAG

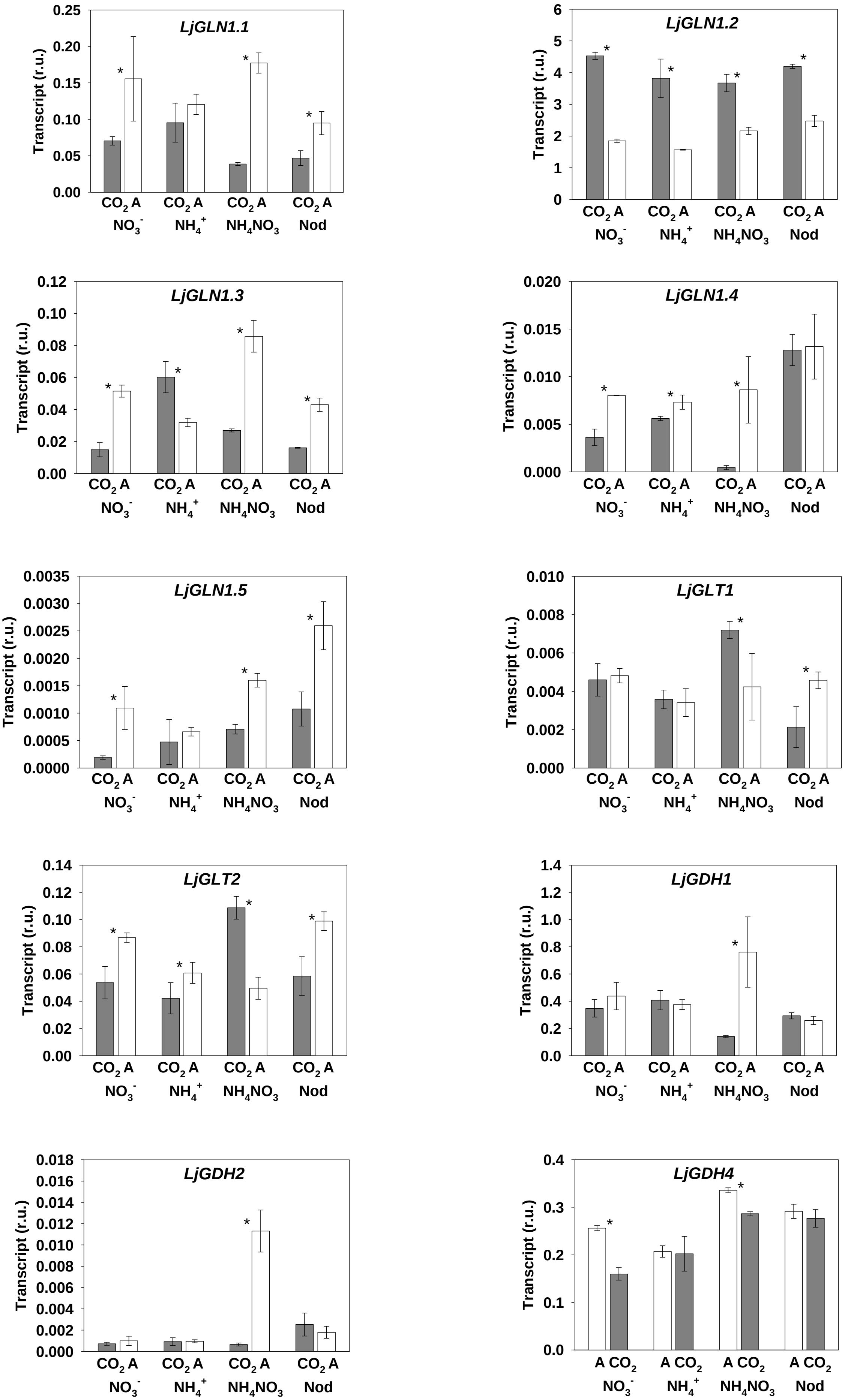

LjGLN1.1
Transcript (r.u.)
0.25
0.20
0.15
0.10
0.05
0.00
CO2 A
NO3-
NH4+
NH4NO3
Nod
LjGLN1.2
6
5
4
3
2
1
0
LjGLN1.3
0.12
0.10
0.08
0.06
0.04
0.02
0.00
LjGLN1.4
0.020
0.015
0.010
0.005
0.000
LjGLN1.5
0.0035
0.0030
0.0025
0.0020
0.0015
0.0010
0.0005
0.0000
LjGLT1
0.010
0.008
0.006
0.004
0.002
0.000
LjGLT2
0.14
0.12
0.10
0.08
0.06
0.04
0.02
0.00
LjGDH1
1.4
1.2
1.0
0.8
0.6
0.4
0.2
0.0
LjGDH2
0.018
0.016
0.014
0.012
0.010
0.008
0.006
0.004
0.002
0.000
LjGDH4
0.4
0.3
0.2
0.1
0.0
A CO2

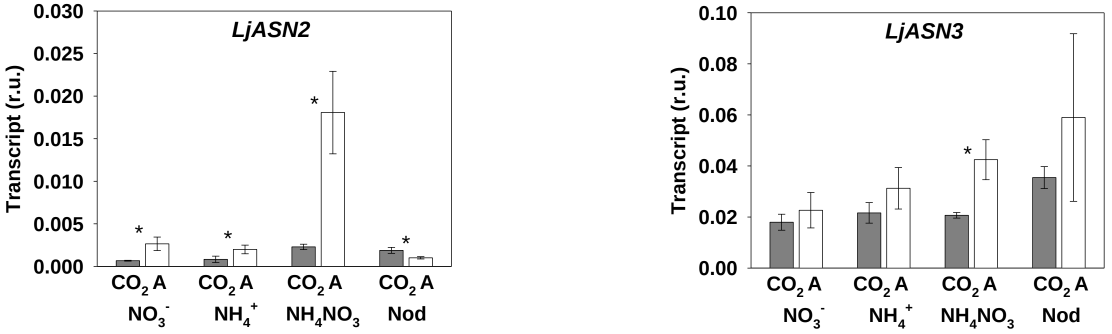

Supplementary Fig. S1. Expression levels of some key genes of nitrogen metabolism in WT plants under CO_2-enriched atmosphere (CO_2, grey bars) or normal air (A, white bars) and different nitrogen sources. *LjGLN*: cytosolic glutamine synthetase; *LjGLT*: NADH-glutamate synthase; *LjGDH*: glutamate dehydrogenase; *LjASN*: asparagine synthetase. *Indicates significant difference between both conditions determined by student's test ($P < 0.05$).

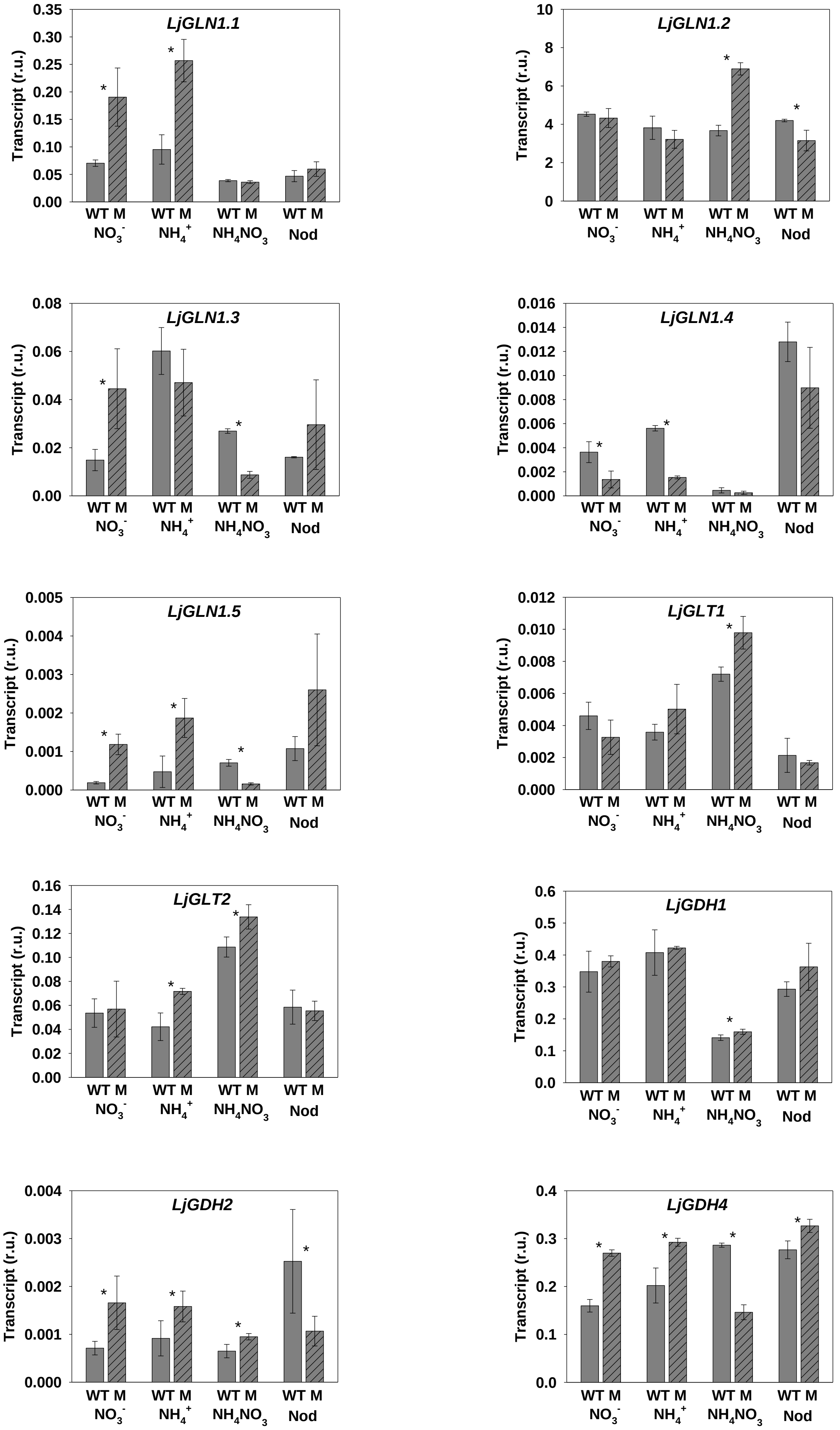

LjGLN1.1
LjGLN1.2
LjGLN1.3
LjGLN1.4
LjGLN1.5
LjGLT1
LjGLT2
LjGDH1
LjGDH2
LjGDH4
Transcript (r.u.)
WT M
NO3-
NH4+
NH4NO3
Nod
*

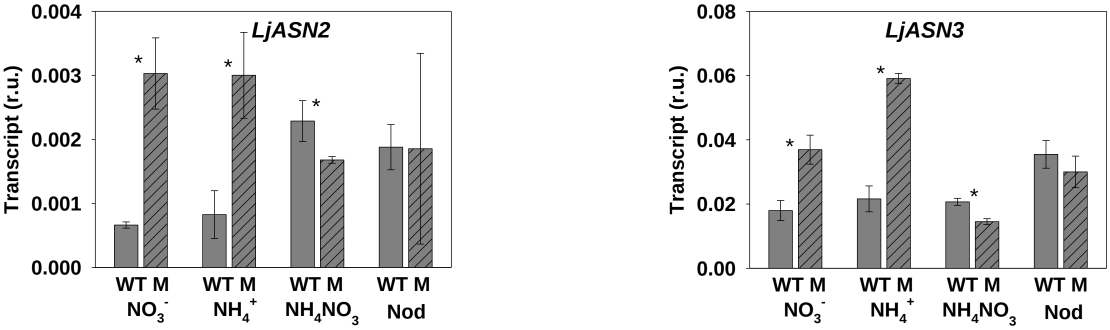

Supplementary Fig. S2. Expression levels of some key genes of nitrogen metabolism in WT (WT, grey bars) and *Ljgln2-2* plants (M, grey bars filled with stripes) grown under different nitrogen nutrition and under CO_2-enriched atmosphere. *LjGLN*: cytosolic glutamine synthetase; *LjGLT*: NADH-glutamate synthase; *LjGDH*: glutamate dehydrogenase; *LjASN*: asparagine synthetase. *Indicates significant difference between WT and *Ljgln2-2* determined by student's test ($P < 0.05$).

WT -WTair
(A)

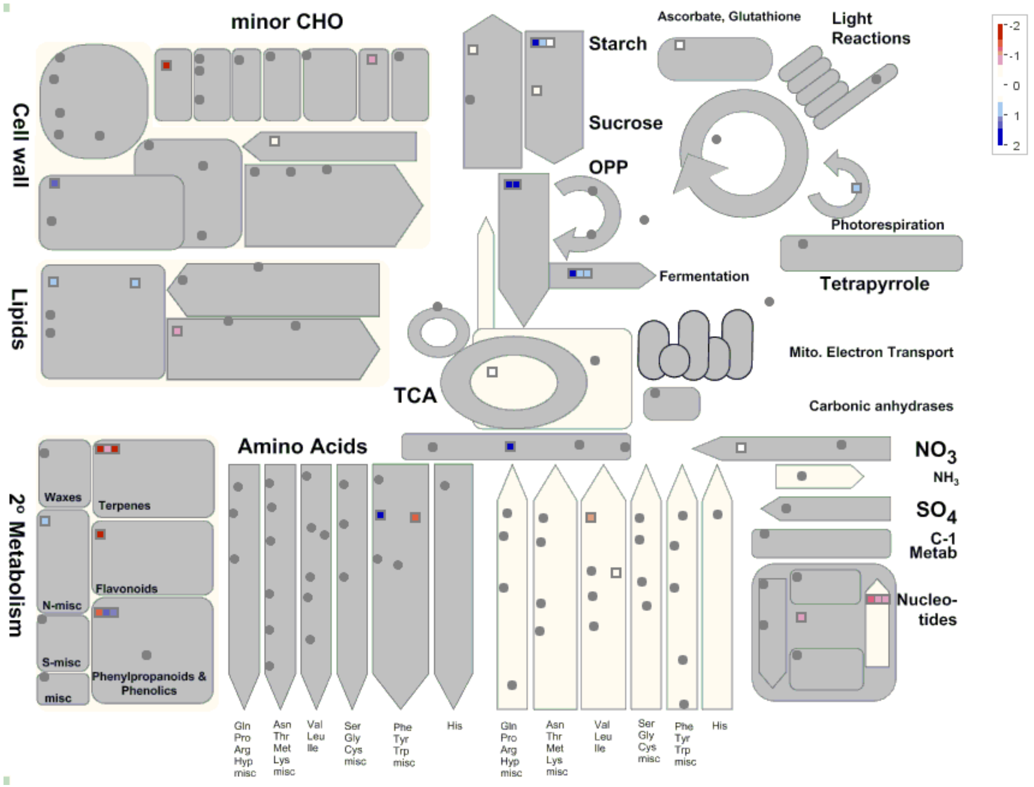

***Ljgln2-2* - WT**
(B)

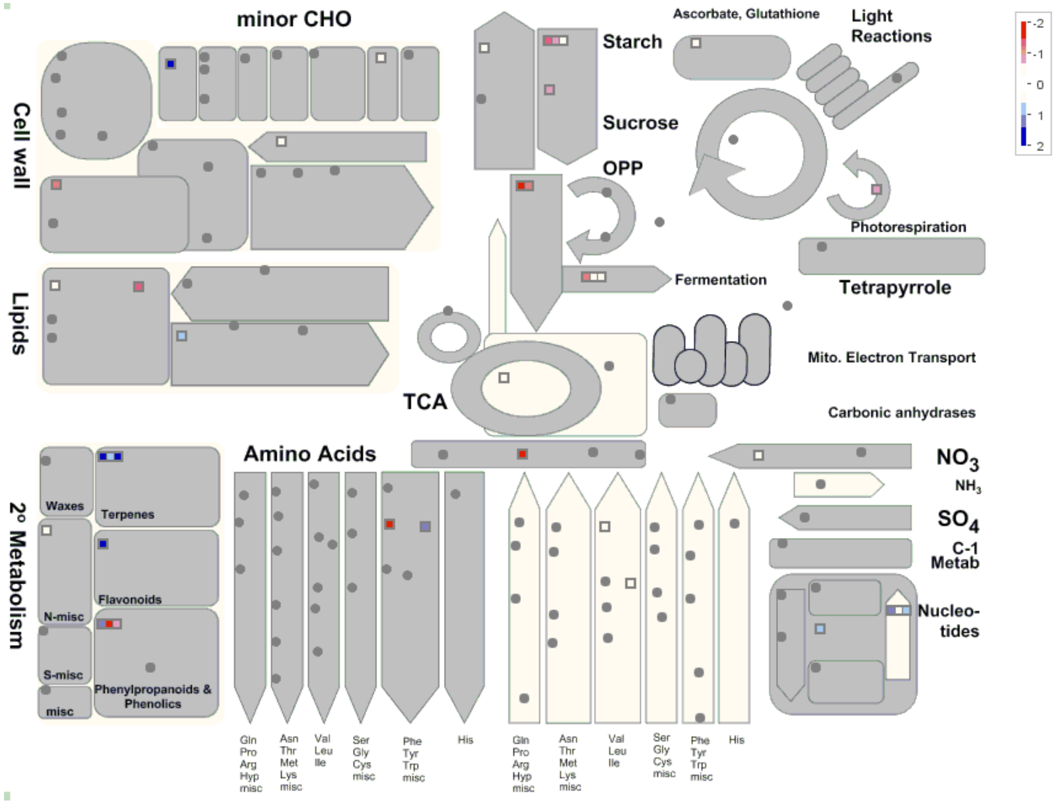

Pathway			Nb. of Enzymes submitted
Name		Nb. of Enzymes	
Flavonoid biosynthesis	map	15	5
Histidine metabolism	map	16	5
D-Arginine and D-ornithine metabolism	map	2	2
Starch and sucrose metabolism	map	31	7
Valine, leucine and isoleucine degradation	map	15	4

Supplementary Fig. S3. MapMan metabolism overview and Pathexpress analysis of over-represented pathways of genes modulated by the diminishment of CO_2 concentration and by the absence of plastidic GS (288 probesets). (A) MapMan metabolism overview of these 288 probesets differentially expressed in leaves of plants grown under CO_2-enriched atmosphere compared to leaves of plants grown under normal air atmosphere. (B) MapMan metabolism overview of these 288 probesets differentially expressed in leaves of *Ljgln2-2* plants compared to leaves of WT plants under CO_2-enriched atmosphere. Changes in gene expression were analyzed by Rank Product applying a false discovery rate ($FDR < 0.1$). Each square corresponds to a gene. Red and blue indicate lower and higher expression than the control, respectively. The scale bar is shown in $\log_2$.
